# Supplementary material for: Neural cell state modulation by PARK2 and dopaminergic neuroprotection by small molecule Parkin agonism
Source: bioRxiv. 2026 Apr 4:2026.04.01.715918. Preprint. [Version 1] doi: 10.64898/2026.04.01.715918 (PMC13060259; doi:10.64898/2026.04.01.715918)
Supplement: 1 [file NIHPP2026.04.01.715918v1-supplement-1.pdf]

bioRxiv preprint doi: https://doi.org/10.64898/2026.04.01.715918; this version posted April 4, 2026. The copyright holder for this preprint (which was not certified by peer review) is the author/funder, who has granted bioRxiv a license to display the preprint in perpetuity. It is made available under aCC-BY 4.0 International license.

Supplementary Figure Legends

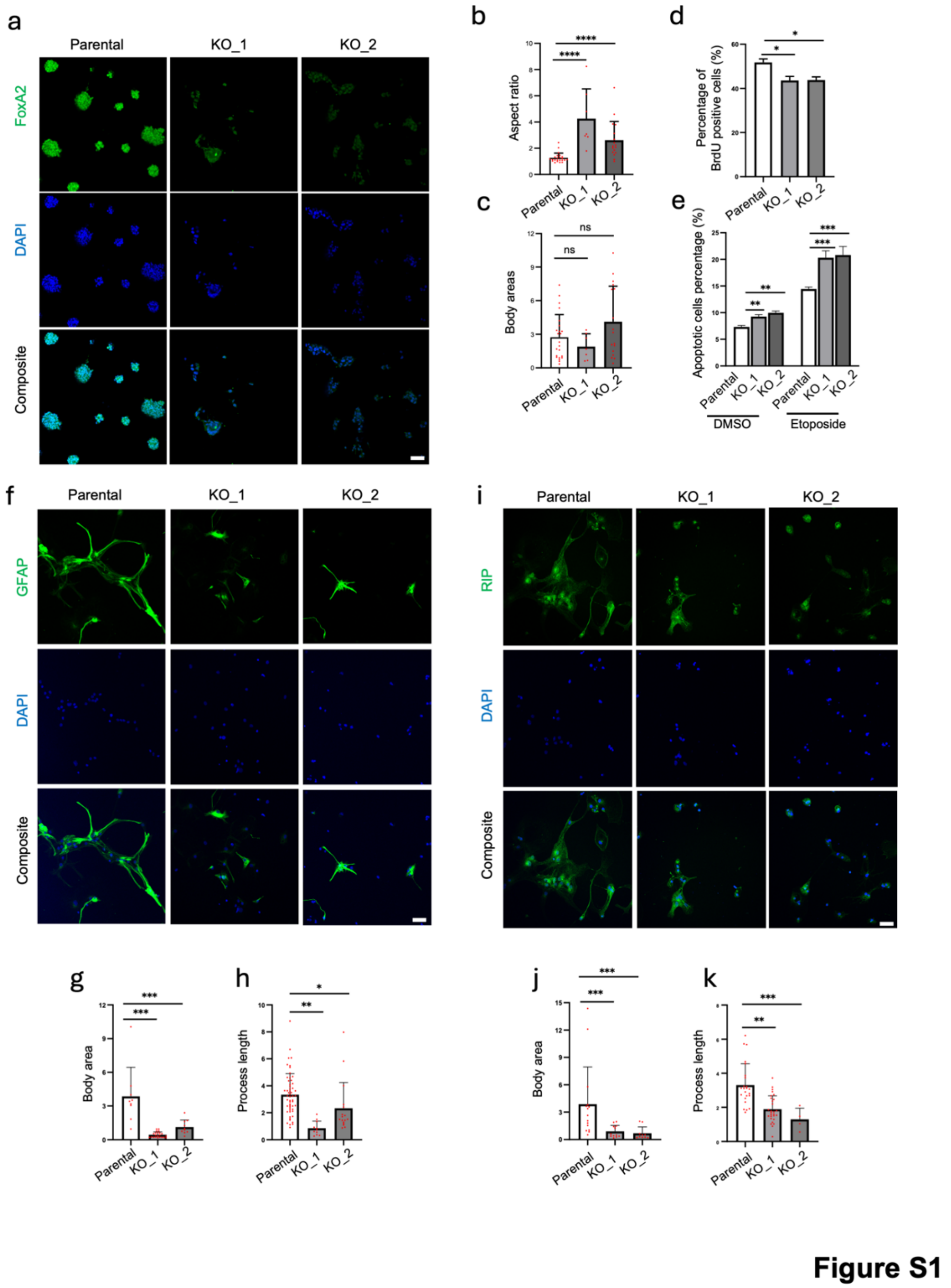

Supplementary Figure 1. Parkin KO results in phenotypic abnormalities in NP cells.
(**a**) Immunofluorescence staining of FoxA2 in Parkin KO and parental (WT) NPC cells. Parkin KO disrupts the normal shape and structure of cell bodies and cell aggregation. Scale bar = 50 µm. (**b**) Quantitation of average aspect ratios and (**c**) cell body sizes. (**d**) Flow cytometry shows Parkin KO NPCs have a decreased number of cells in S phase. (**e**) Flow cytometry shows Parkin KO NPC cells have an increase of apoptotic cells both at baseline and after etoposide treatment. (**f**) Immunofluorescence staining of GFAP (marker of astrocytes) in differentiated Parkin KO and parental cells. Quantification of astrocyte phenotypes showing (**g**) difference in body area between the genotypes and (**h**) differences in average process length. (**i**) Immunofluorescence

bioRxiv preprint doi: https://doi.org/10.64898/2026.04.01.715918; this version posted April 4, 2026. The copyright holder for this preprint (which was not certified by peer review) is the author/funder, who has granted bioRxiv a license to display the preprint in perpetuity. It is made available under aCC-BY 4.0 International license.

staining of RIP (marker of oligodendrocytes) in differentiated Parkin KO and parental cells. Quantitation of oligodendrocyte phenotypes showing **(j)** difference in body area between the genotypes and (**k**) differences in average process length. All plots show individual data points, the mean, and SD. ANOVA and post-hoc Tukey's test or student's t-test (for f). *p<0.05, **p<0.01, ***p<0.001. ****p<0.0001. ns=not significant.

bioRxiv preprint doi: https://doi.org/10.64898/2026.04.01.715918; this version posted April 4, 2026. The copyright holder for this preprint (which was not certified by peer review) is the author/funder, who has granted bioRxiv a license to display the preprint in perpetuity. It is made available under aCC-BY 4.0 International license.

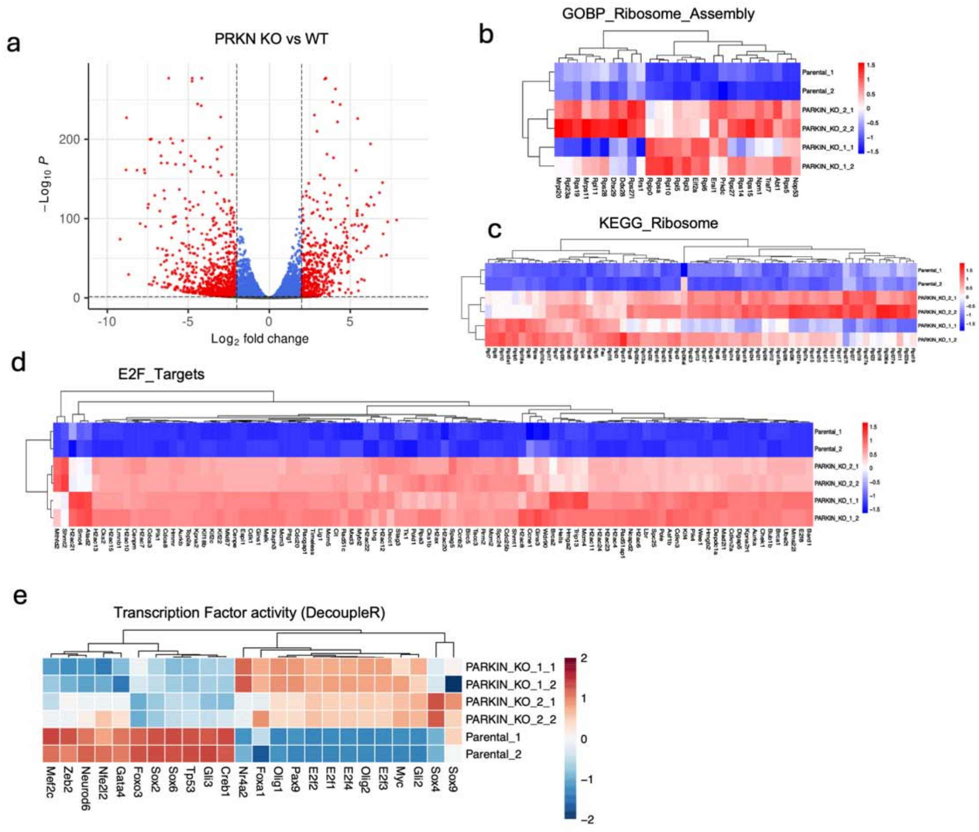

Figure S2

Supplementary Figure 2. Parkin KO-induced changes in the transcriptome.
(**a**) Gene expression changes in Parkin KO neural progenitor cells. Volcano plot showing differentially expressed genes comparing KO (n=4) and WT (n=2) samples. Crosshairs show cutoffs for adjusted p-values <0.05 and Log2FC >2 or <-2. Red dots represent those values which met our threshold of p-adjusted values <0.05 and Log2FC >2 or <-2; blue and grey dots indicate not significant. (**b-d**) Examples of gene expression programs undergoing large-scale changes due to Parkin inactivation. GSEA analysis of differentially expressed genes between KO vs WT cells showing leading edge genes for pathways of GOBP Ribosome assembly, KEGG Ribosome, and E2F targets. Data are plotted in heatmaps with gene values scaled across samples.

bioRxiv preprint doi: https://doi.org/10.64898/2026.04.01.715918; this version posted April 4, 2026. The copyright holder for this preprint (which was not certified by peer review) is the author/funder, who has granted bioRxiv a license to display the preprint in perpetuity. It is made available under aCC-BY 4.0 International license.

(**e**) Differential transcription factor activity in Parkin KO versus WT cells. DecoupleR was used to identify pathway activity. Pathways with top differential activity scores are shown.

bioRxiv preprint doi: https://doi.org/10.64898/2026.04.01.715918; this version posted April 4, 2026. The copyright holder for this preprint (which was not certified by peer review) is the author/funder, who has granted bioRxiv a license to display the preprint in perpetuity. It is made available under aCC-BY 4.0 International license.

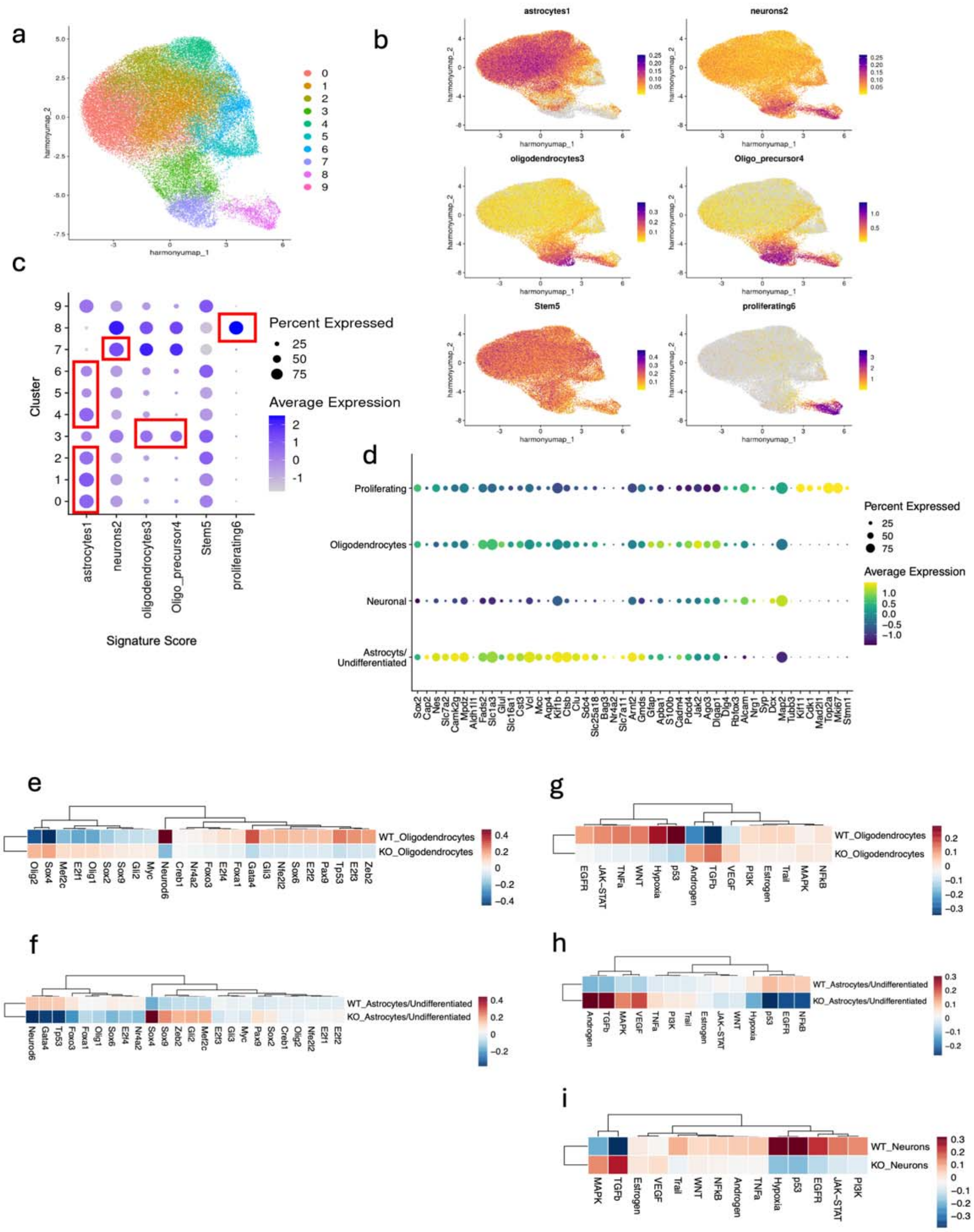

Figure S3

Supplementary Figure 3. Single-cell sequencing of differentiated Parkin KO neural and glial cells reveals lineage-specific gene expression defects.

(**a**) UMAP showing clusters identified in differentiated cells. Unsupervised clustering was performed using Seurat, with Harmony batch correction to generate a single harmonized UMAP with 10 clusters. n=2 KO and n=3 WT Parkin clones. (**b**) Gene set overlays identify astrocytes, neurons/neuron-like cells, oligodendrocytes, oligodendrocyte precursors, stem cells, and proliferating cells. Data generated and scored using Seurat's AddModuleScore function. Gene set scores are shown as feature plots, highlighted where the gene set of interest is most highly enriched on the UMAP. (**c**) Four primary classes of differentiated cells were identified and related to their corresponding clusters: astrocytes, neuronal, oligodendrocytes, and proliferating

bioRxiv preprint doi: https://doi.org/10.64898/2026.04.01.715918; this version posted April 4, 2026. The copyright holder for this preprint (which was not certified by peer review) is the author/funder, who has granted bioRxiv a license to display the preprint in perpetuity. It is made available under aCC-BY 4.0 International license.

(marked with red boxes). (**d**) Key cell lineage markers across primary classes of differentiated cells. After naming the 4 cellular classes by grouping clusters into similarly scored groups, key genes distinguishing each class are shown by gene. For each dot, expression level is represented by the color scale and size of each dot represents the percentage of cells expressing a given gene. (**e, f**) Altered neurodevelopmental-related transcription factor activity in Parkin KO cells. DecoupleR transcription factor analysis of Parkin KO vs WT oligodendrocytes and astrocytes/undifferentiated cells. (**g-i**) DecoupleR pathway activity analysis of Parkin KO vs WT cells. Neuronal, oligodendrocyte, and astrocytes/undifferentiated cells are shown, highlighting the top-most altered pathways.

bioRxiv preprint doi: https://doi.org/10.64898/2026.04.01.715918; this version posted April 4, 2026. The copyright holder for this preprint (which was not certified by peer review) is the author/funder, who has granted bioRxiv a license to display the preprint in perpetuity. It is made available under aCC-BY 4.0 International license.

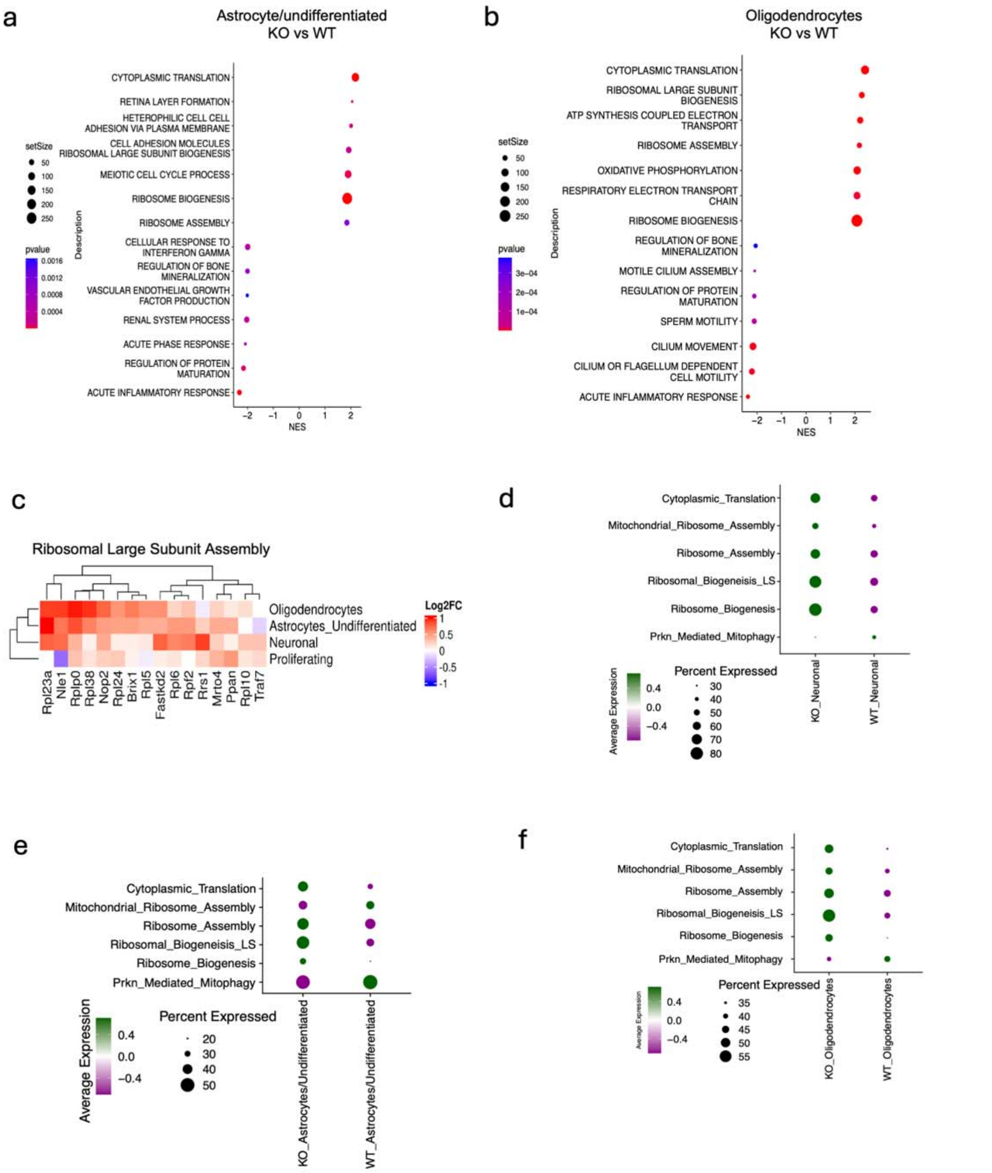

Figure S4

Supplementary Figure 4. Gene expression programs altered in Parkin KO astrocytes and oligodendrocytes.

(**a**) Differential gene expression of Parkin KO vs WT astrocyte/undifferentiated cells. Data shows output of GSEA analysis using the GOBP gene set. Most enriched pathways are shown. NES, normalized enrichment score. Dot size represents the input gene set size, and color scale shows the p-value. (**b**) Differential gene expression of Parkin KO vs oligodendrocytes. Data shows output of GSEA analysis using the GOBP gene set. Most enriched pathways shown. NES, normalized enrichment score. Dot size represents the input gene set size, and color scale shows the p-value. (**c**) High expression of ribosome assembly genes in Parkin KO cells. Differential expression was performed for each cell type, and the log2FC value of genes from the ribosomal

bioRxiv preprint doi: https://doi.org/10.64898/2026.04.01.715918; this version posted April 4, 2026. The copyright holder for this preprint (which was not certified by peer review) is the author/funder, who has granted bioRxiv a license to display the preprint in perpetuity. It is made available under aCC-BY 4.0 International license.

large subunit assembly pathway from GOBP were plotted. Color scale shows the log2FC value of each comparison. (**d-f**) Relative expression of gene sets for ribosomal pathways, translation, and mitophagy are shown. Seurat's AddModuleScore was used to score individual cells across the entire dataset for each gene set. After scoring cells, the labelled cell types for each genotype were plotted, where dot features indicate the average expression score of each gene set and the percent of cells expressing the gene set.

bioRxiv preprint doi: https://doi.org/10.64898/2026.04.01.715918; this version posted April 4, 2026. The copyright holder for this preprint (which was not certified by peer review) is the author/funder, who has granted bioRxiv a license to display the preprint in perpetuity. It is made available under aCC-BY 4.0 International license.

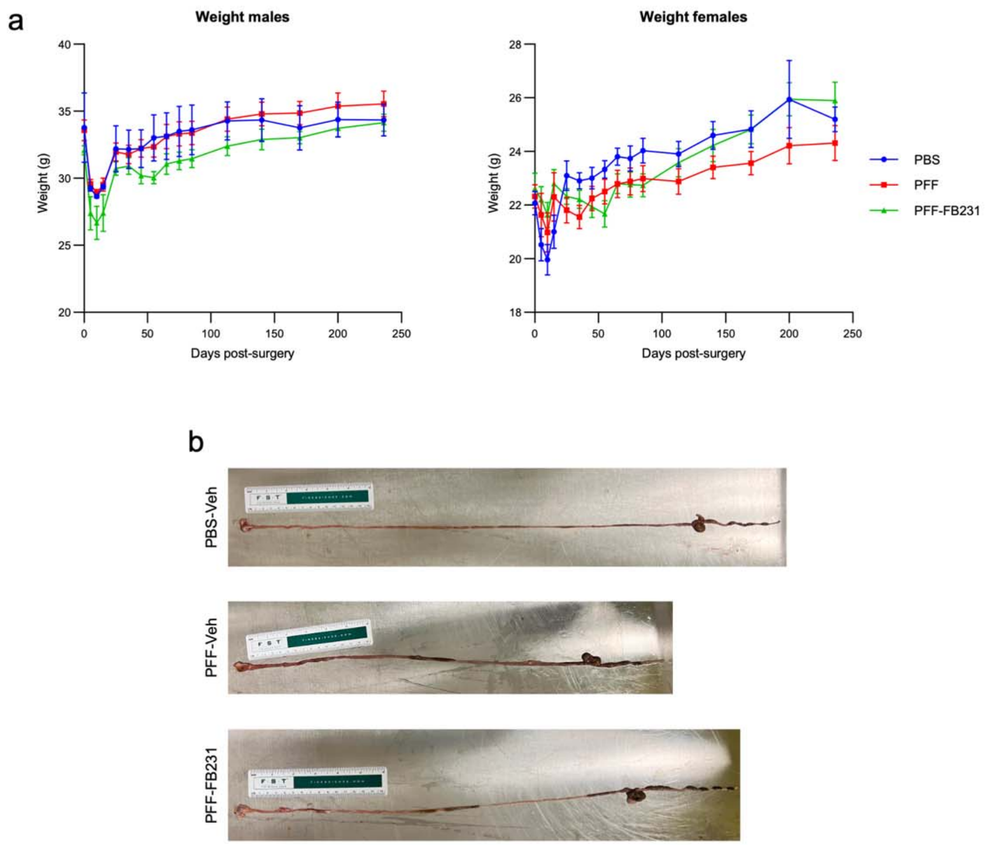

Figure S5

Supplementary Figure 5. Mice body weight and GI length measurement

(a) The body weight of the mice used in our experiments was monitored over 8 months across the different experimental conditions for both male and female mice. n=6-16 mice/group. (b) Representative GI length measurement of mice in different experimental groups.

bioRxiv preprint doi: https://doi.org/10.64898/2026.04.01.715918; this version posted April 4, 2026. The copyright holder for this preprint (which was not certified by peer review) is the author/funder, who has granted bioRxiv a license to display the preprint in perpetuity. It is made available under aCC-BY 4.0 International license.

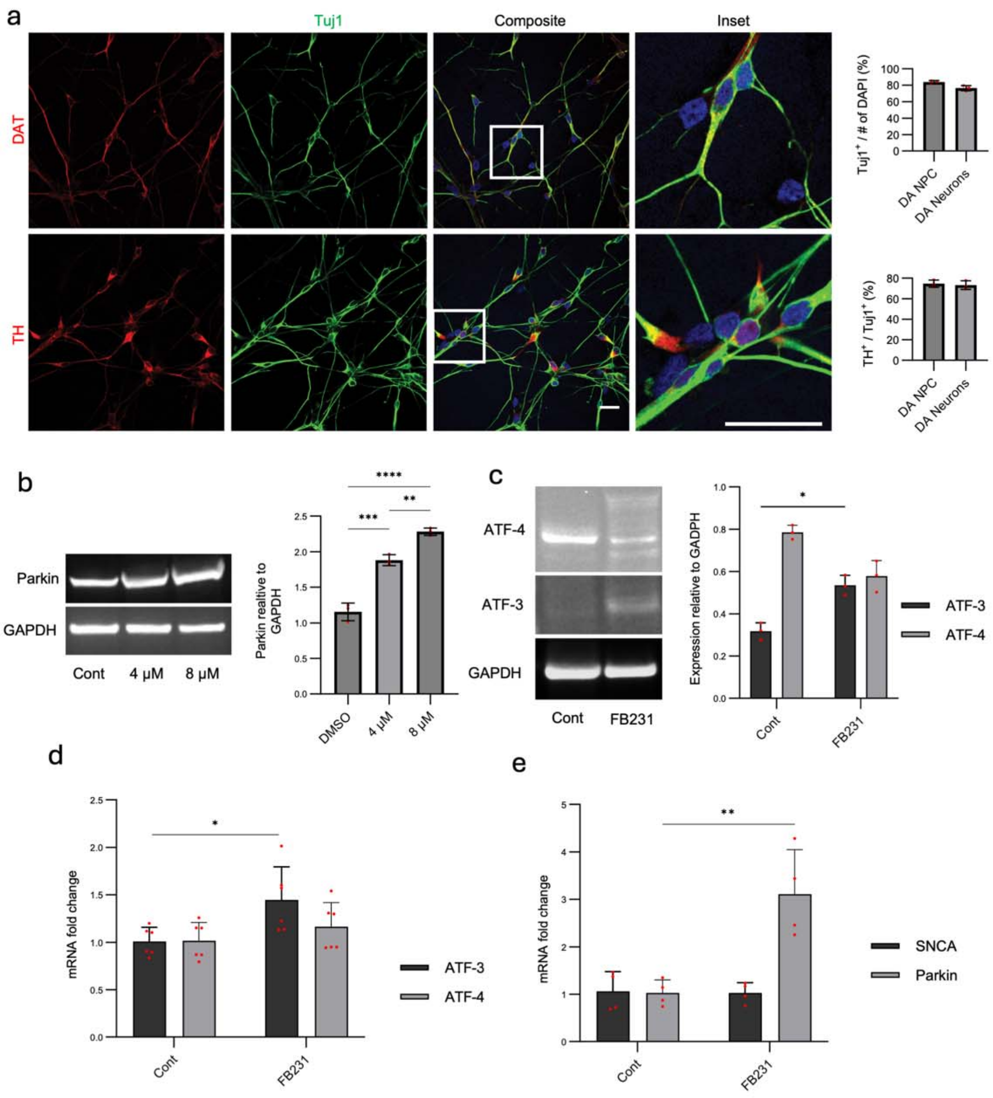

Figure S6

bioRxiv preprint doi: https://doi.org/10.64898/2026.04.01.715918; this version posted April 4, 2026. The copyright holder for this preprint (which was not certified by peer review) is the author/funder, who has granted bioRxiv a license to display the preprint in perpetuity. It is made available under aCC-BY 4.0 International license.

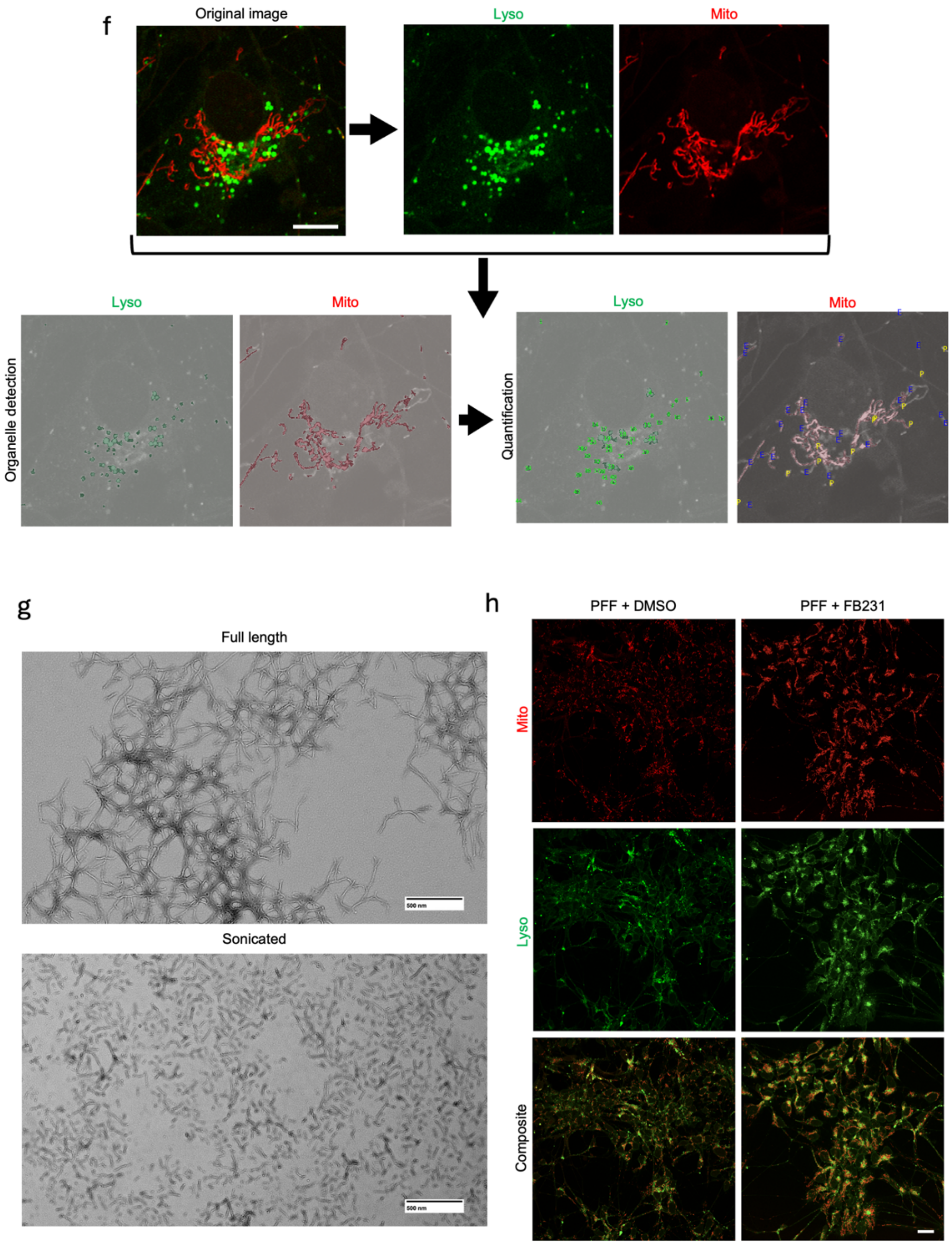

Supplementary Figure 6. Characterization of iPSC-derived dopaminergic neurons, FB231-upregulation of mitophagy, and PFFs.

(**a**) TH and DAT staining in matured iPSC-derived dopaminergic neurons used to assess the efficiency of dopaminergic neuron generation. Quantification of Tuj1-positive neurons divided by the total number of cells (DAPI: 4,6-diamidino-2-phenylindole) yielded the percentage of neuronal cells per imaging field. Scale bar = 20 µm. Additionally, the quantification of TH-positive neurons divided by the total number of neurons (number of Tuj1-positive) yielded the percentage of dopaminergic neurons per imaging field. (**b**) Parkin upregulation in healthy iPSC-derived dopaminergic neurons following 48 h of incubation with 4 µM and 8 µM FB231 or DMSO (Cont). GAPDH was used as a loading control. (**c**) FB231 (4 µM) and DMSO-treated

bioRxiv preprint doi: https://doi.org/10.64898/2026.04.01.715918; this version posted April 4, 2026. The copyright holder for this preprint (which was not certified by peer review) is the author/funder, who has granted bioRxiv a license to display the preprint in perpetuity. It is made available under aCC-BY 4.0 International license.

dopaminergic neurons were harvested 36 hours after treatment for Western blot and other experiments. (**d** and **e**) qPCR analysis of the samples used in **c**. (**f**) Analysis pipeline used for detecting and quantifying organelle morphology. Scale bar = 10 µm. (**g**) Transmission electron microscopy (TEM) characterization of recombinant PFFs before and after sonication, via fibril length. (**h**) Large-field Mitotracker (Mito) and Lysotracker (Lyso) staining and colocalization in neurons previously exposed to PFFs at 1 µg/mL for 24 h, then exposed to 4µM FB231 or DMSO for 24 h. Scale bar = 20 µm.

bioRxiv preprint doi: https://doi.org/10.64898/2026.04.01.715918; this version posted April 4, 2026. The copyright holder for this preprint (which was not certified by peer review) is the author/funder, who has granted bioRxiv a license to display the preprint in perpetuity. It is made available under aCC-BY 4.0 International license.

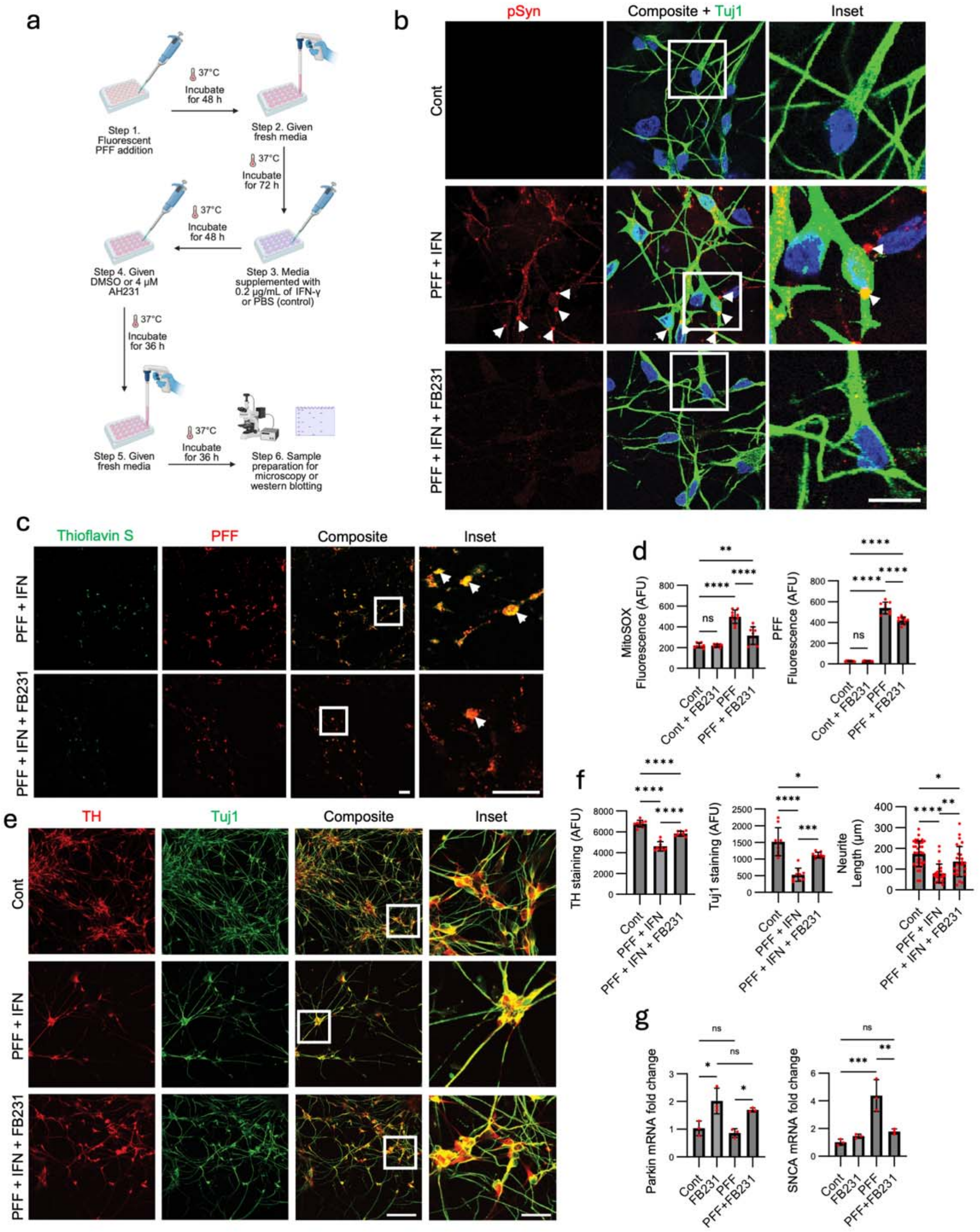

Figure S7

Supplementary Figure 7. The neuroprotective effects of FB231 in neurons exposed to PFF and IFN-γ.

(**a**) Outline of the adapted dual hit treatment regime used in this study. (**b**) Dopaminergic neurons underwent the dual hit treatment regimen for 10 days and then were stained for pSyn. PFF + IFN-γ-treated conditions exhibited high levels of pSyn, with several larger punctae indicating αSyn-positive inclusions (arrowhead). However, when IFN-γ exposure was followed by exposure to FB231, pSyn fluorescence was dramatically reduced. Scale bar = 10 μm. (**c**) Thioflavin S staining of PFF-positive inclusions in PFF + IFN-γ and PFF + IFN-γ + FB231 samples. Scale bar = 20 μm. (**d**) Unbiased quantification of PFF and MitoSOX fluorescence, along with TH and Tuj1 fluorescence, was done for additional experimental and control groups,

bioRxiv preprint doi: https://doi.org/10.64898/2026.04.01.715918; this version posted April 4, 2026. The copyright holder for this preprint (which was not certified by peer review) is the author/funder, who has granted bioRxiv a license to display the preprint in perpetuity. It is made available under aCC-BY 4.0 International license.

including control samples treated with FB231, along with PFF-only samples with/without FB231 treatment. (**e**) Neuronal survival and neurite morphology assessed by TH and Tuj1 immunostaining. Scale bar = 100 µm and 25 µm for insets. (**f**) Unbiased quantification of TH and Tuj1 fluorescence and neurite length measured by NeuronJ plugin. (**g**) qPCR analysis of the samples visualized in **e**. All plots show individual data points, the mean, and SD. One-way ANOVA and *post-hoc* Tukey's test were used for statistical analysis. *p<0.05, **p<0.01, ***p<0.001, ****p<0.001; "ns", not statistically significant.
